# Supplementary material for: Pyruvate Dehydrogenase Kinase 1 inhibition mediated oxidative phosphorylation enhancement in cartilage promotes osteoarthritis progression
Source: BMC Musculoskelet Disord. 2023 Jul 20;24:597. doi: 10.1186/s12891-023-06585-6 (PMC10357736; doi:10.1186/s12891-023-06585-6)
Supplement: Supplementary file 1 — Additional file 1. [file 12891_2023_6585_MOESM1_ESM.zip › BP.Gsea.1653623667859/GOBP_RESPONSE_TO_ATP.html]

Details for gene set GOBP\_RESPONSE\_TO\_ATP[GSEA]

|  || Dataset | 55457\_collapsed\_to\_symbols.cls.cls #OA\_versus\_normal.cls.cls #OA\_versus\_normal\_repos |
| Phenotype | cls.cls#OA\_versus\_normal\_repos |
| Upregulated in class | normal |
| GeneSet | GOBP\_RESPONSE\_TO\_ATP |
| Enrichment Score (ES) | -0.51991916 |
| Normalized Enrichment Score (NES) | -1.7310786 |
| Nominal p-value | 0.0034188034 |
| FDR q-value | 0.12343345 |
| FWER p-Value | 1.0 |
Table: GSEA Results Summary

  

Fig 1: Enrichment plot: GOBP\_RESPONSE\_TO\_ATP      
 Profile of the Running ES Score & Positions of GeneSet Members on the Rank Ordered List

  

| SYMBOL | TITLE | RANK IN GENE LIST | RANK METRIC SCORE | RUNNING ES | CORE ENRICHMENT || 1 | P2RX4 | purinergic receptor P2X 4 [Source:HGNC Symbol;Acc:HGNC:8535] | 1827 | 0.381 | -0.1027 | No |
| 2 | SOD1 | superoxide dismutase 1 [Source:HGNC Symbol;Acc:HGNC:11179] | 2413 | 0.313 | -0.1160 | No |
| 3 | DGKQ | diacylglycerol kinase theta [Source:HGNC Symbol;Acc:HGNC:2856] | 2516 | 0.302 | -0.0929 | No |
| 4 | P2RX5 | purinergic receptor P2X 5 [Source:HGNC Symbol;Acc:HGNC:8536] | 2937 | 0.257 | -0.0991 | No |
| 5 | P2RX2 | purinergic receptor P2X 2 [Source:HGNC Symbol;Acc:HGNC:15459] | 4029 | 0.153 | -0.1682 | No |
| 6 | P2RX7 | purinergic receptor P2X 7 [Source:HGNC Symbol;Acc:HGNC:8537] | 4415 | 0.120 | -0.1857 | No |
| 7 | P2RY11 | purinergic receptor P2Y11 [Source:HGNC Symbol;Acc:HGNC:8540] | 4936 | 0.077 | -0.2182 | No |
| 8 | TRPM4 | transient receptor potential cation channel subfamily M member 4 [Source:HGNC Symbol;Acc:HGNC:17993] | 5667 | 0.018 | -0.2731 | No |
| 9 | TRPV1 | transient receptor potential cation channel subfamily V member 1 [Source:HGNC Symbol;Acc:HGNC:12716] | 6032 | -0.007 | -0.3006 | No |
| 10 | CIB2 | calcium and integrin binding family member 2 [Source:HGNC Symbol;Acc:HGNC:24579] | 7311 | -0.103 | -0.3893 | No |
| 11 | PANX1 | pannexin 1 [Source:HGNC Symbol;Acc:HGNC:8599] | 7344 | -0.105 | -0.3810 | No |
| 12 | HSP90B1 | heat shock protein 90 beta family member 1 [Source:HGNC Symbol;Acc:HGNC:12028] | 9134 | -0.242 | -0.4950 | Yes |
| 13 | ABCC9 | ATP binding cassette subfamily C member 9 [Source:HGNC Symbol;Acc:HGNC:60] | 9346 | -0.259 | -0.4848 | Yes |
| 14 | P2RY2 | purinergic receptor P2Y2 [Source:HGNC Symbol;Acc:HGNC:8541] | 9470 | -0.271 | -0.4664 | Yes |
| 15 | SSH1 | slingshot protein phosphatase 1 [Source:HGNC Symbol;Acc:HGNC:30579] | 9748 | -0.296 | -0.4575 | Yes |
| 16 | P2RY1 | purinergic receptor P2Y1 [Source:HGNC Symbol;Acc:HGNC:8539] | 9925 | -0.314 | -0.4389 | Yes |
| 17 | P2RX1 | purinergic receptor P2X 1 [Source:HGNC Symbol;Acc:HGNC:8533] | 9926 | -0.314 | -0.4066 | Yes |
| 18 | P2RY4 | pyrimidinergic receptor P2Y4 [Source:HGNC Symbol;Acc:HGNC:8542] | 9978 | -0.319 | -0.3778 | Yes |
| 19 | PKLR | pyruvate kinase L/R [Source:HGNC Symbol;Acc:HGNC:9020] | 10120 | -0.333 | -0.3545 | Yes |
| 20 | RYR3 | ryanodine receptor 3 [Source:HGNC Symbol;Acc:HGNC:10485] | 10344 | -0.355 | -0.3353 | Yes |
| 21 | P2RX3 | purinergic receptor P2X 3 [Source:HGNC Symbol;Acc:HGNC:8534] | 11903 | -0.572 | -0.3975 | Yes |
| 22 | TRPC3 | transient receptor potential cation channel subfamily C member 3 [Source:HGNC Symbol;Acc:HGNC:12335] | 11922 | -0.577 | -0.3396 | Yes |
| 23 | IL1B | interleukin 1 beta [Source:HGNC Symbol;Acc:HGNC:5992] | 12105 | -0.622 | -0.2898 | Yes |
| 24 | SLC8A1 | solute carrier family 8 member A1 [Source:HGNC Symbol;Acc:HGNC:11068] | 12192 | -0.642 | -0.2305 | Yes |
| 25 | P2RX6 | purinergic receptor P2X 6 [Source:HGNC Symbol;Acc:HGNC:8538] | 12529 | -0.762 | -0.1782 | Yes |
| 26 | PTGS2 | prostaglandin-endoperoxide synthase 2 [Source:HGNC Symbol;Acc:HGNC:9605] | 12641 | -0.820 | -0.1026 | Yes |
| 27 | TAF1 | TATA-box binding protein associated factor 1 [Source:HGNC Symbol;Acc:HGNC:11535] | 12881 | -1.192 | 0.0015 | Yes |
Table: GSEA details [plain text format]

  

Fig 2: GOBP\_RESPONSE\_TO\_ATP      
 Blue-Pink O' Gram in the Space of the Analyzed GeneSet

  

Fig 3: GOBP\_RESPONSE\_TO\_ATP: Random ES distribution      
 Gene set null distribution of ES for **GOBP\_RESPONSE\_TO\_ATP**
